# The Effect of Internet-Delivered Cognitive Behavioral Therapy Versus Psychoeducation Only on Psychological Distress in Patients With Noncardiac Chest Pain: Randomized Controlled Trial

**DOI:** 10.2196/31674

**Published:** 2022-01-28

**Authors:** Ghassan Mourad, Magda Eriksson-Liebon, Patric Karlström, Peter Johansson

**Affiliations:** 1 Department of Health, Medicine and Caring Sciences Linköping University Linköping Sweden; 2 Department of Emergency Medicine in Norrköping, and Department of Biomedical and Clinical Sciences, Linköping University Linköping Sweden; 3 Department of Internal Medicine, Ryhov County Hospital, Region Jönköping County Jönköping Sweden; 4 Department of Internal Medicine in Norrköping, and Department of Health, Medicine and Caring Sciences, Linköping University Linköping Sweden

**Keywords:** cardiac anxiety, cognitive behavioral therapy, health-related quality of life, internet delivered, noncardiac chest pain, psychological distress

## Abstract

**Background:**

Patients with recurrent episodes of noncardiac chest pain (NCCP) experience cardiac anxiety as they misinterpret the pain to be cardiac related and avoid physical activity that they think could threaten their lives. Psychological interventions, such as internet-delivered cognitive behavioral therapy (iCBT), targeting anxiety can be a feasible solution by supporting patients to learn how to perceive and handle their chest pain.

**Objective:**

This study aims to evaluate the effects of a nurse-led iCBT program on cardiac anxiety and other patient-reported outcomes in patients with NCCP.

**Methods:**

Patients with at least two health care consultations because of NCCP during the past 6 months, and who were experiencing cardiac anxiety (Cardiac Anxiety Questionnaire score ≥24), were randomized into 5 weeks of iCBT (n=54) or psychoeducation (n=55). Patients were aged 54 (SD 17) years versus 57 (SD 16) years and were mainly women (32/54, 59% vs 35/55, 64%). The iCBT program comprised psychoeducation, mindfulness, and exposure to physical activity, with weekly homework assignments. The primary outcome was cardiac anxiety. The secondary outcomes were fear of bodily sensations, depressive symptoms, health-related quality of life, and chest pain frequency. Intention-to-treat analysis was applied, and the patients were followed up for 3 months. Mixed model analysis was used to determine between-group differences in primary and secondary outcomes.

**Results:**

No significant differences were found between the iCBT and psychoeducation groups regarding cardiac anxiety or any of the secondary outcomes in terms of the interaction effect of time and group over the 3-month follow-up. iCBT demonstrated a small effect size on cardiac anxiety (Cohen *d*=0.31). In the iCBT group, 36% (16/44) of patients reported a positive reliable change score (≥11 points on the Cardiac Anxiety Questionnaire), and thus an improvement in cardiac anxiety, compared with 27% of (13/48) patients in the psychoeducation group. Within-group analysis showed further significant improvement in cardiac anxiety (*P*=.04) at the 3-month follow-up compared with the 5-week follow-up in the iCBT group but not in the psychoeducation group.

**Conclusions:**

iCBT was not superior to psychoeducation in decreasing cardiac anxiety in patients with NCCP. However, iCBT tends to have better long-term effects on psychological distress, including cardiac anxiety, health-related quality of life, and NCCP frequency than psychoeducation. The effects need to be followed up to draw more reliable conclusions.

**Trial Registration:**

ClinicalTrials.gov NCT03336112; https://www.clinicaltrials.gov/ct2/show/NCT03336112

## Introduction

### Background

Approximately 50% of patients seeking acute care because of chest pain have noncardiac chest pain (NCCP) [1-5], which is equivalent to approximately 80,000 people annually in Sweden [6]. Despite recurrent chest pain, many patients are discharged without a clear explanation regarding the underlying cause of their chest pain [7,8], and a large proportion of them continue to experience chest pain [8-10]. Wertli et al [11], in a retrospective study of 1341 emergency department admissions among patients with NCCP, concluded that there is an uncertainty among physicians regarding how to approach these patients after ruling out the cardiac causes. In fact, many of these patients are still not convinced by their negative cardiac diagnosis several years after the primary assessment, and they worry about heart disease, avoid activities that they think might be harmful to their heart, and repeatedly seek medical help [12,13]. Physical activity has been shown to be associated with a lower risk of experiencing NCCP. Therefore, avoidance of such activities has negative effects on these patients [14]. This situation seems to cause functional impairment, psychological distress, and impaired quality of life in these patients [4,15-18]. Studies have also suggested that patients with NCCP use outpatient health care to the same extent as patients with myocardial infarction [4,17,19] and incur high costs for the health care system and society [1,6,20-22].

Cardiac anxiety has been found to be highly prevalent in these patients and is strongly associated with health care use [9,18]. An explanation is that cardiac anxiety may worsen the chest pain and create a *vicious circle*, leading to the maintenance of both anxiety and chest pain. Psychological interventions targeting anxiety can be a feasible way of breaking the vicious circle and improving patient outcomes by supporting patients in learning how to perceive and handle their chest pain. This is also warranted by the World Health Organization, which emphasizes the need for the implementation of interventions targeting psychological distress in patients with somatic diseases [23].

There is strong support for cognitive behavioral therapy (CBT) in the treatment of mild and moderately severe states of anxiety and depressive disorders [24-27]. CBT is a structured and collaborative process aiming to help individuals evaluate the accuracy and usefulness of their thoughts [28]. A Cochrane review found CBT useful and moderately successful, despite the multifaceted etiology in NCCP, and stressed the need for further randomized controlled trials of psychological interventions for NCCP with long follow-up periods [29]. However, a problem is that there are some barriers to the use of face-to-face CBT [30]. In addition to being time consuming, there is a lack of trained health care professionals who can deliver CBT, leading to a treatment demand gap; thus, the number of patients in need of CBT is higher than the number being offered CBT [29,31]. A possible solution is to use the internet to deliver CBT (internet-delivered CBT [iCBT]), as it does not differ from face-to-face CBT regarding the effects among young and middle-aged people [32-35], requires less therapist involvement, can be given to more patients, is cheaper, and is not time or room dependent [30].

However, few studies have evaluated the effects and experiences of iCBT in patients with both somatic disease and psychological distress [36,37]. However, in a recent study, iCBT provided by nurses, with a brief introduction in iCBT, to patients with cardiac disease and depressive symptoms was shown to reduce depressive symptoms and improve the quality of life [38]. This implies that health care professionals, such as nurses within somatic health care, could use iCBT as a tool for treating patients, which could increase the access to CBT for psychological distress among patients with somatic diseases, such as NCCP. Furthermore, no studies have been undertaken concerning patients with NCCP, apart from our previous pilot study that tested the feasibility of iCBT in patients with NCCP and cardiac anxiety. Our piloted iCBT program was perceived as feasible by the patients [39]. Thus, there is a knowledge gap regarding the effects of iCBT programs guided by health care professionals, such as nurses, on cardiac anxiety and other patient-reported outcomes in patients with NCCP.

### Objective

The aim of this study is to evaluate the effects of a nurse-led iCBT program on cardiac anxiety and other patient-reported outcomes in patients with NCCP.

## Methods

### Design and Ethics

This was a randomized controlled trial and was registered at ClinicalTrials.gov (NCT03336112). The study was approved by the regional ethical review board in Linköping, Sweden (code 2017/343-31).

### Study Participants

Patients were eligible for participation if they were aged ≥18 years, had at least two health care consultations because of NCCP (International Classification of Diseases-10 codes R07.2, R07.3, R07.4, and Z03.4) during the past 6 months, and screened positive for cardiac anxiety (score ≥24 on the Cardiac Anxiety Questionnaire [CAQ]). Patients were excluded if they had no access to a computer or tablet with internet connection; were not able to perform physical activity because of physical constraints; did not speak or understand Swedish; or experienced severe depression, as measured by the Patient Health Questionnaire-9 (PHQ-9), cognitive impairment, or newly diagnosed cancer requiring treatment (according to medical records).

### Procedures

Study participants were recruited after discharge from the emergency units at 3 regional hospitals and 1 university hospital in southeast Sweden. Recruitment of patients to the study was conducted between January 2018 and August 2020. At the start of the study, we only recruited patients from 2 hospitals; however, to allow for faster recruitment, we expanded to 2 more hospitals from April 2019. Eligible participants were identified by registers and sent a packet, which included study information, an informed consent form, and a prestamped envelope by post. After some days, patients were contacted by phone by the study team and informed verbally about the study. Patients interested in participation and who sent back a signed written informed consent form were screened for cardiac anxiety using an encrypted web-based survey tool provided by Linköping University and requiring both username and password to log in. Patients fulfilling the criteria were randomized in a 1:1 ratio into either iCBT or psychoeducation using a randomization table provided by a statistician. Masking of patients was not possible as the intervention was a CBT program. Participating in the study (in both the iCBT and psychoeducation groups) did not oppose seeking or receiving other inpatient or outpatient treatments.

The intervention was delivered through a website specifically developed for the study by the first author (GM). The website was accessible only to those who received the website URL and log-in details. In addition to username and password, 2-factor authentication was applied using an SMS text messaging one-time password to ensure security.

### iCBT Group

The iCBT group received a 5-session/week–long nurse-led iCBT program that was developed based on our previous pilot study [39]. This pilot study contained 4 sessions and was extended by 1 week to give patients more time to work on the content for better effects. The program comprised psychoeducation, mindfulness, and exposure to physical activity, with weekly homework assignments. The psychoeducation part was aimed at teaching patients about chest pain and its impact on daily life and how avoidance and safety behaviors can maintain or exacerbate chest pain. A chest pain diary and exposure plan were set up by the patients to help them learn about their chest pain and not be afraid of exposing themselves to physical activity that many patients normally avoid. The mindfulness part, comprising both information and different exercises to perform daily, was intended to raise awareness of what is going on in the body, emotions, and sensations and to be more in the present despite chest pain to learn how to handle the chest pain. The physical activity part comprised information and recommendations regarding physical activity based on national guidelines [40]. The goal of this part was to enable patients to learn that their hearts tolerate physical activity and to reduce cardiac anxiety and avoidance of physical activity.

The patients had various weekly assignments to accomplish and send in for feedback. They then received feedback and advice from the same nurse once per week at the same time to allow them to plan their time and engagement in the program. The participants were fully aware of this procedure. The entire treatment was conducted through the study website. Reminders and encouraging messages were sent to motivate the patients to complete the program. The iCBT program accounted for the main part of the treatment. The guidance and feedback part took approximately a mean of 8 (SD 4) minutes per patient and week.

### Psychoeducation Group

Patients with NCCP can be seen as a forgotten group as they usually have no regular follow-ups regarding their chest pain. Thus, their care as usual is equivalent to no care at all. Therefore, comparing iCBT with care as usual is equivalent to comparing with nothing, and therefore, the effect of iCBT could be overestimated, as it would be difficult to evaluate if the effect only depended on iCBT or partly on the attention of being in a study [41]. It is also recommended that control patients in iCBT studies receive an active control [36]. Furthermore, it is, for ethical reasons, better to offer patients with different problems some kind of intervention than nothing at all when randomizing them to the control arm [41]. On the basis of this, our active control group received psychoeducation (ie, containing the same information that the iCBT group received as part of their psychoeducation but without any assignments or feedback). This information was divided into 5 sessions, and patients accessed 1 session per week through the same website as the iCBT group. The active control group received a message from the therapist every week when a new session was made available.

### Data Collection

Data were collected at baseline before randomization; at the end of the intervention (ie, 5 weeks from baseline), and at 3, 6, and 12 months after the end of the intervention. In this study, data from the 6- and 12-month follow-ups were not included. Medical data were provided via medical records, and the rest were self-reported. All self-reported data were collected using the encrypted web-based survey tool provided by Linköping University. As all questions were mandatory to be able to submit the questionnaires, this resulted in no missing values among those who chose to answer the questionnaires. In total, participants received 2 reminders every 2 weeks if they did not complete the questionnaires.

The primary outcome was cardiac anxiety. The secondary outcomes were fear of bodily sensations, depressive symptoms, health-related quality of life (HRQoL), chest pain frequency, health care use, and health care costs. Data on health care use and health care costs will be published separately.

### Data Measurement

#### Cardiac Anxiety

Cardiac anxiety was measured by the CAQ. This measurement comprises 18 items, with scores ranging from 0 to 72. Higher scores indicate greater cardiac anxiety [42]. In this study, we used a median score of 24 from one of our previous studies [18] as the cutoff score for cardiac anxiety. In addition to the total score obtained from the 18 items, the CAQ comprises three subscales: fear, avoidance, and heart-focused attention. However, these subscales are not presented in this study. The total scale has demonstrated adequate reliability and validity [42]. Cronbach α coefficients of .79 to .89 were confirmed in this study at the different measurement points.

#### Fear of Bodily Sensations

The Body Sensations Questionnaire (BSQ) was used to assess the fear of bodily sensations, such as palpitations, dizziness, and sweating. The BSQ comprises 17 items, with scores between 17 and 85. Higher scores indicate greater fear of bodily sensations [43]. The BSQ has been proven to be reliable and valid [13,18,43]. In this study, the Cronbach α coefficients were .92 to .94.

#### Depressive Symptoms

The prevalence and severity of depressive symptoms were assessed using the PHQ-9. This measurement comprises 9 items, with a score range between 0 and 27. Scores between 5 and 9 indicate mild depressive symptoms, 10 and 14 indicate moderate depressive symptoms, 15 and 19 indicate moderately severe depressive symptoms, and 20 and 27 indicate severe depressive symptoms. In this study, we used a cutoff score of 10 for depressive symptoms. The PHQ-9 is a reliable instrument [44], and in this study, the Cronbach α coefficients were .87 to .89.

#### Chest Pain Frequency

Data on chest pain frequency were collected with the following self-developed question: *During the last month, how often have you experienced NCCP?*

#### HRQoL Measure

The EuroQol visual analog scale (EQ-VAS), which is a part of EuroQol, was used to measure HRQoL. This EQ-VAS scores range between 0 (worst imaginable health state) and 100 (best imaginable health state). The EQ-VAS provides a quantitative measure of the patient’s perception of their overall health and is a frequently used instrument [45].

### Statistical Analysis

The SPSS (version 25; IBM Corp) was used for data analysis. The level of *P*<.05 was set for significance. According to our power calculation that was based on results from our previous pilot study [39], 53 participants needed to be included in each group to reach a 20% improvement (approximately an effect size of 0.5) in cardiac anxiety (95% CI and 80% power). This was used as there is no established score for a clinically significant improvement in this measurement. This sample size is comparable with similar CBT studies [32]. Intention-to-treat analysis was applied, and patients were followed up even if they were inactive or dropped out from the study. This method was chosen to preserve the integrity of the randomization and minimize the risks of bias related to differences in groups following attrition or nonadherence [46].

Frequencies, percentages, mean values, and SDs were used to describe and compare the study variables. Chi-square test or 2-tailed Student *t* test was used depending on the level of the data to compare differences between iCBT and psychoeducation groups regarding demographic variables.

To compare the iCBT and psychoeducation groups regarding changes in cardiac anxiety, bodily sensations, depressive symptoms, HRQoL, and NCCP frequency over the 3 different measurement points (ie, baseline and 5-week and 3-month follow-ups), a mixed model analysis was performed. As we had little missing data (in total, 8% at 5-week follow-up and 16% at 3-month follow-up), we chose to base our analysis on the original data; however, we also ran a mixed model analysis based on multiple imputation as a sensitivity analysis to ensure the accuracy of our results. Multiple imputation was based on the consideration of data being missing at random (the Little missing completely at random test; *P*=.99) [47]. According to this method, missing values depend on the observed data and are replaced by values based on the complete data set [48]. In this model, a total of 40 imputations were calculated based on the outcome and demographic variables that showed a significant correlation with the primary outcome at baseline. The results from the mixed model analysis based on multiple imputation did not differ from the analysis that was based on the original data; therefore, these results are not presented in this study.

Relationships between demographic variables, disease and psychological burden, treatment activity (such as number of log-ins and sessions performed), and change score in cardiac anxiety between baseline and 3-month follow-up in the groups were determined using the Pearson correlation coefficient. As there were no significant correlations, no further regression analysis was performed.

Cohen *d* was used to measure the effect sizes of the intervention. Effect sizes <0.20 were considered trivial, 0.20 to 0.49 were considered small, 0.50 to 0.79 were considered moderate, and ≥0.80 were considered large [49]. As there is no established score for a clinically significant improvement in CAQ, we calculated a reliable change index score according to the study by Christensen et al [50] using the baseline SD of 8.6 and baseline reliability score of 0.79 in our groups. On the basis of these values, a reliable change was deemed to be a change score of approximately 11 points per participant. The chi-square test was used to examine the difference between groups in the number of patients with a change score ≥11.

Within-group differences over the 3 different measurement points were analyzed using paired *t* tests.

## Results

### Study Participants

**Figure 1:**
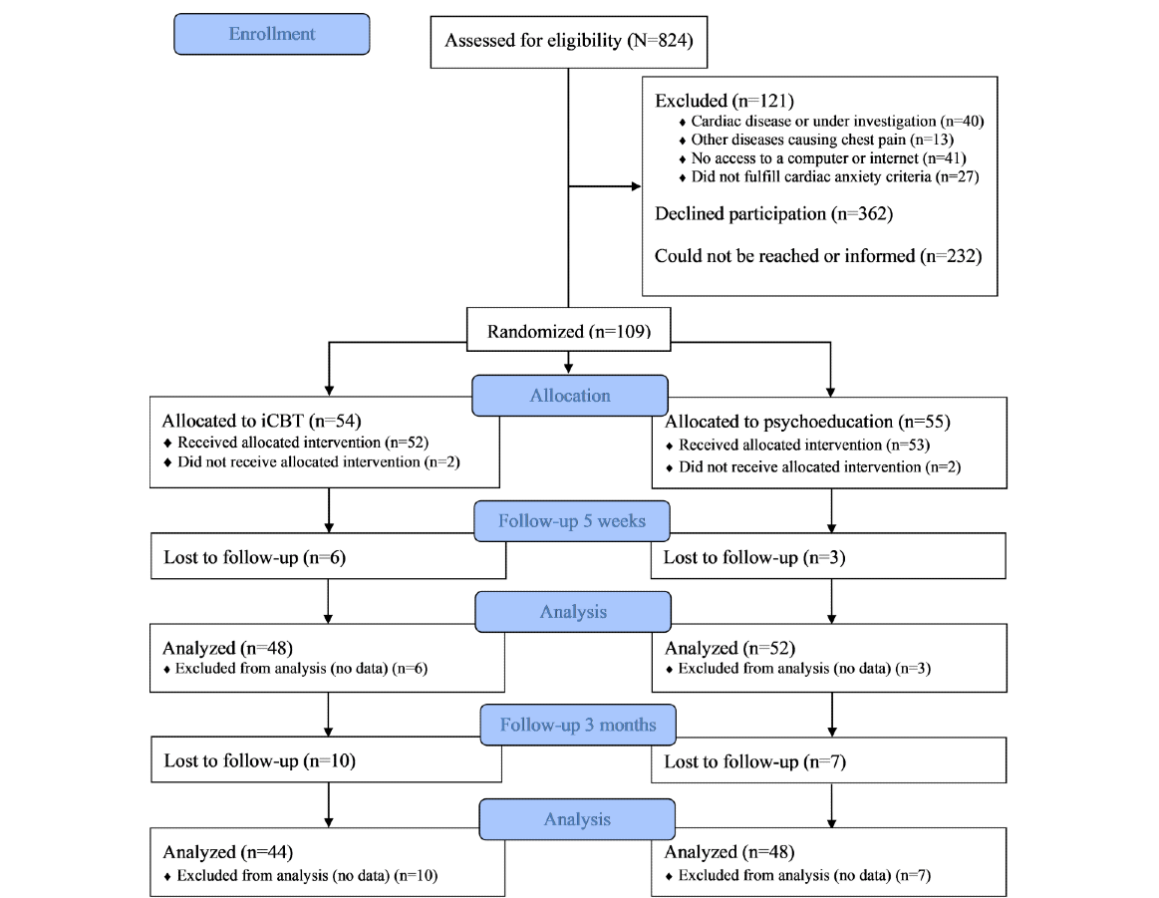
The CONSORT (Consolidated Standards of Reporting Trials) flowchart. iCBT: internet-delivered cognitive behavioral therapy.

In total, 824 patients were assessed for eligibility and invited to participate in the study (Figure 1). After screening, 13.2% (109/824) of patients who fulfilled the inclusion and exclusion criteria and consented to participate were randomized into iCBT (54/109, 49.5%) or psychoeducation (55/109, 50.4%). Study participants included a higher proportion of women than nonparticipants (715/824, 86.8%; 67/109, 61.5% vs 354/715, 49.5%; *P*=.02).

The demographic data for the iCBT and psychoeducation groups are presented in Table 1.

Patients in these groups had a mean age of 54 (17) years versus 57 (SD 16) years and were mainly women (32/54, 59% vs 35/55, 64%) and in a relationship (45/54, 83% vs 38/55, 69%). Approximately 33% (18/54) in the iCBT group and 27% (15/55) in the psychoeducation group had previous heart disease, 43% (23/54) in the iCBT group, and 34% (19/55) in the psychoeducation group reported previous problems with psychological distress (such as anxiety, panic, or depression), and more than half reported troubles with musculoskeletal pain. Furthermore, approximately half of them (iCBT 23/54, 43% vs psychoeducation 25/55, 45%) had been treated with psychotropic drugs. No significant differences were found between the groups for any of the demographic variables.

Among the participants, the attrition rates at the 5-week follow-up were 11% and 5% for the iCBT and psychoeducation groups, respectively. Patients in the iCBT group who were lost to the 5-week follow-up, that is, did not fill in the questionnaires, were more often on sick leave (*P*=.02). In addition, they had low or no engagement in the program; that is, they completed a mean of 1 session compared with 4.2 in those who continued their participation (*P*<.001). In the psychoeducation group, those lost to follow-up were more often single and living alone (*P*=.008). As for the iCBT group, those in the psychoeducation group who were lost to the 5-week follow-up had low or no engagement in the program, that is, completed a mean of 0.33 sessions compared with 4.0 in those who continued their participation (*P*<.001). The corresponding attrition rates of iCBT and psychoeducation groups for the 3-month follow-up were 19% and 13%, respectively, compared with baseline. The only differences between those who continued their participation and those lost to the 3-month follow-up was a lower number of completed sessions in the latter group (a mean of 4.1 vs 2.8 sessions, *P*=.02, in the iCBT group and a mean of 4.2 vs 1.6 sessions, *P*<.001, in the psychoeducation group).

**Table 1:** Demographic data of study patients at baseline (N=109).

Characteristics			iCBT^a^ (n=54)		Psychoeducation (n=55)		*P* value	
Age (year), mean (SD)			54.3 (16.5)		56.8 (15.5)		.42	
**Sex, n (%)**								.64
	Women	32 (59)		35 (64)				
	Men	22 (41)		20 (36)				
**Marital status, n (%)**								.08
	In a relationship	45 (83)		38 (69)				
	Single	9 (17)		17 (31)				
Salary (US $), mean (SD)			3564 (5742)		4018 (5486)		.69	
**Economic situation, n (%)**								.46
	Very good	5 (9)		7 (13)				
	Good	42 (78)		38 (69)				
	Problematic	7 (13)		10 (18)				
**Educational level, n (%)**								.89
	Compulsory school	7 (13)		8 (15)				
	High school	23 (43)		25 (45)				
	University	24 (44)		22 (40)				
**Occupational status, n (%)**								.30
	Working	20 (37)		27 (49)				
	Retired	20 (37)		19 (35)				
	On sick leave	6 (11)		5 (9)				
	Unemployed	2 (4)		3 (5)				
	Student	6 (11)		1 (2)				
**Smoking, n (%)**								.97
	Nonsmoker or previous smoker	49 (91)		48 (87)				
	Smoker	5 (9)		7 (13)				
**Alcohol consumption, n (%)**								.58
	Never or seldom	28 (52)		25 (45)				
	≤9 glasses/week	24 (44)		30 (55)				
	>9 glasses/week	2 (4)		0 (0)				
Exercise ≥30 minutes (days/week), mean (SD)			2.5 (2.1)		3.0 (2.2)		.24	
**Origin, n (%)**								.36
	Sweden	45 (85)		42 (76)				
	Another Nordic country	2 (4)		3 (6)				
	Another country within Europe	5 (9)		5 (9)				
	South America	1 (2)		1 (2)				
	Asia	0 (0)		4 (7)				
Charlson Comorbidity Index, mean (SD)			2.2 (2.4)		2.5 (2.1)		.57	
Previous heart disease, n (%)			18 (33)		15 (27)		.49	
Acid reflux, n (%)			9 (17)		9 (16)		.97	
Muscle pain, n (%)			29 (54)		26 (47)		.50	
Joint or skeletal pain, n (%)			32 (59)		31 (56)		.76	
Psychological disorder, n (%)			23 (43)		19 (34)		.39	
Psychological treatment, n (%)			8 (15)		5 (9)		.36	
Treatment with psychotropic drugs, n (%)			23 (43)		25 (45)		.76	
Number of log-ins per week, mean (SD)			13.7 (12.8)		5.4 (2.5)		<.001	
**Sessions performed, n (%)**								.06
	0	2 (4)		2 (4)				
	1	5 (9)		4 (7)				
	2	7 (13)		4 (7)				
	3	5 (9)		9 (16)				
	4	1 (2)		10 (18)				
	5	34 (63)		26 (47)				

^a^iCBT: internet-delivered cognitive behavioral therapy.

### The Effects of iCBT Compared With Psychoeducation on Cardiac Anxiety

**Figure 2:**
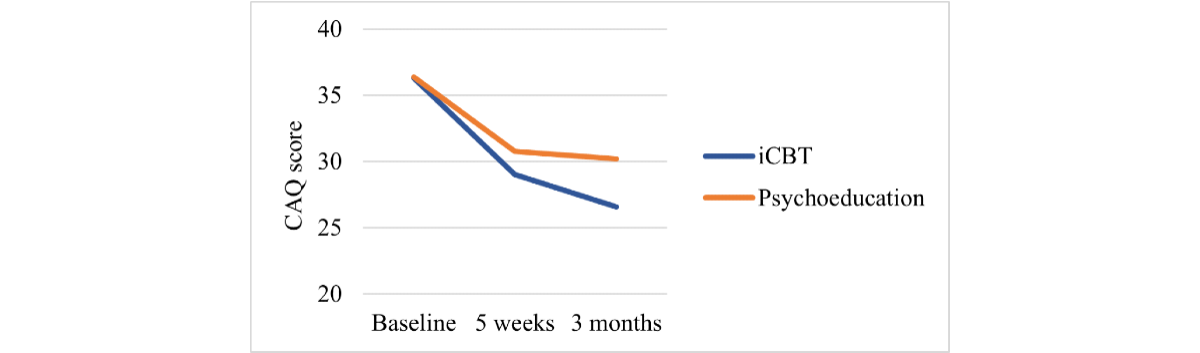
Changes in cardiac anxiety over time between internet-delivered cognitive behavioral therapy (iCBT) and psychoeducation groups. CAQ: Cardiac Anxiety Questionnaire.

The mixed model analysis showed no significant differences between the iCBT and psychoeducation groups regarding cardiac anxiety in terms of the interaction effect of time and group over the 3-month follow-up (Table 2). However, as shown in Figure 2, the iCBT group continued to show improvements in cardiac anxiety at the 3-month follow-up, which was not the case for the psychoeducation group. The analysis showed a small effect size in favor of iCBT (Cohen *d*=0.31). Looking at the time effect, there were statistically significant differences in cardiac anxiety at both the 5-week and 3-month follow-ups compared with baseline (*P*<.001).

In the iCBT group, 36% (16/44) of patients had a positive reliable change score between 11 and 34 points, indicating improvement in cardiac anxiety, whereas 2% (1/44) of patients had a negative reliable change score, showing a deterioration in cardiac anxiety. The corresponding numbers in the psychoeducation group were 27% (13/48) of patients with a positive reliable change score and 2% (1/48) of patients with a negative reliable change score. However, the difference between the groups was nonsignificant (*P*=.21).

Within-group analysis showed that both iCBT and psychoeducation groups improved significantly with respect to cardiac anxiety from baseline to 5-week follow-up (*P*<.001). The iCBT group had an improved mean score from 35.8 to 29.0, whereas the psychoeducation group had an improved score from 36.3 to 30.8. In addition, at the 3-month follow-up, the mean score in the iCBT group was 26.6, which was significantly lower than both baseline (*P*<.001) and the 5-week follow-up (*P*=.04).

**Table 2:** Mixed model analysis of the effect of internet-delivered cognitive behavioral therapy (iCBT) compared with psychoeducation on cardiac anxiety and secondary outcomes, presented in estimated marginal means.

Variables		Time effect		Group effect							Interaction effect				Effect size (Cohen *d*)
		Value, mean	*P* value^a^	iCBT		Psychoeducation		*P* value		iCBT		Psychoeducation	*P* value^a^		
**CAQ^b^**					31.3		32.4		.96						
	Baseline	36.4	N/A^c^							36.3		36.4	N/A	N/A	
	5 weeks	30.3	<.001							29.7		30.8	.46	N/A	
	3 months	28.9	<.001							27.9		29.9	.28	0.31	
**BSQ^d^**					39.3		39.5		.62						
	Baseline	42.7	N/A							43.3		42.1	N/A	N/A	
	5 weeks	38.1	<.001							37.1		39.1	.10	N/A	
	3 months	37.4	<.001							37.5		37.4	.55	0.15	
**PHQ-9^e^**					7.2		6.6		.76						
	Baseline	7.8	N/A							8.0		7.6	N/A	N/A	
	5 weeks	7.0	.54							7.6		6.4	.32	N/A	
	3 months	5.9	.005							6.0		5.8	.87	0.10	
**EQ-VAS^f^**					60.4		63.4		.03						
	Baseline	62.5	N/A							58.5		66.4	N/A	N/A	
	5 weeks	61.4	.49							60.4		62.3	.12	N/A	
	3 months	61.9	.32							62.5		61.5	.10	0.57	
**Chest pain frequency**					13		7.9		.006						
	Baseline	12.0	N/A							15.1		8.9	N/A	N/A	
	5 weeks	11.1	.60							14.1		8.1	.93	N/A	
	3 months	8.3	.005							9.7		6.8	.20	0.21	

^a^In comparison with baseline.

^b^CAQ: Cardiac Anxiety Questionnaire.

^c^N/A: not applicable.

^d^BSQ: Body Sensations Questionnaire.

^e^PHQ-9: Patient Health Questionnaire-9.

^f^EQ-VAS: EuroQol visual analog scale.

### The Effects of iCBT Compared With Psychoeducation on Secondary Outcomes

**Figure 3:**
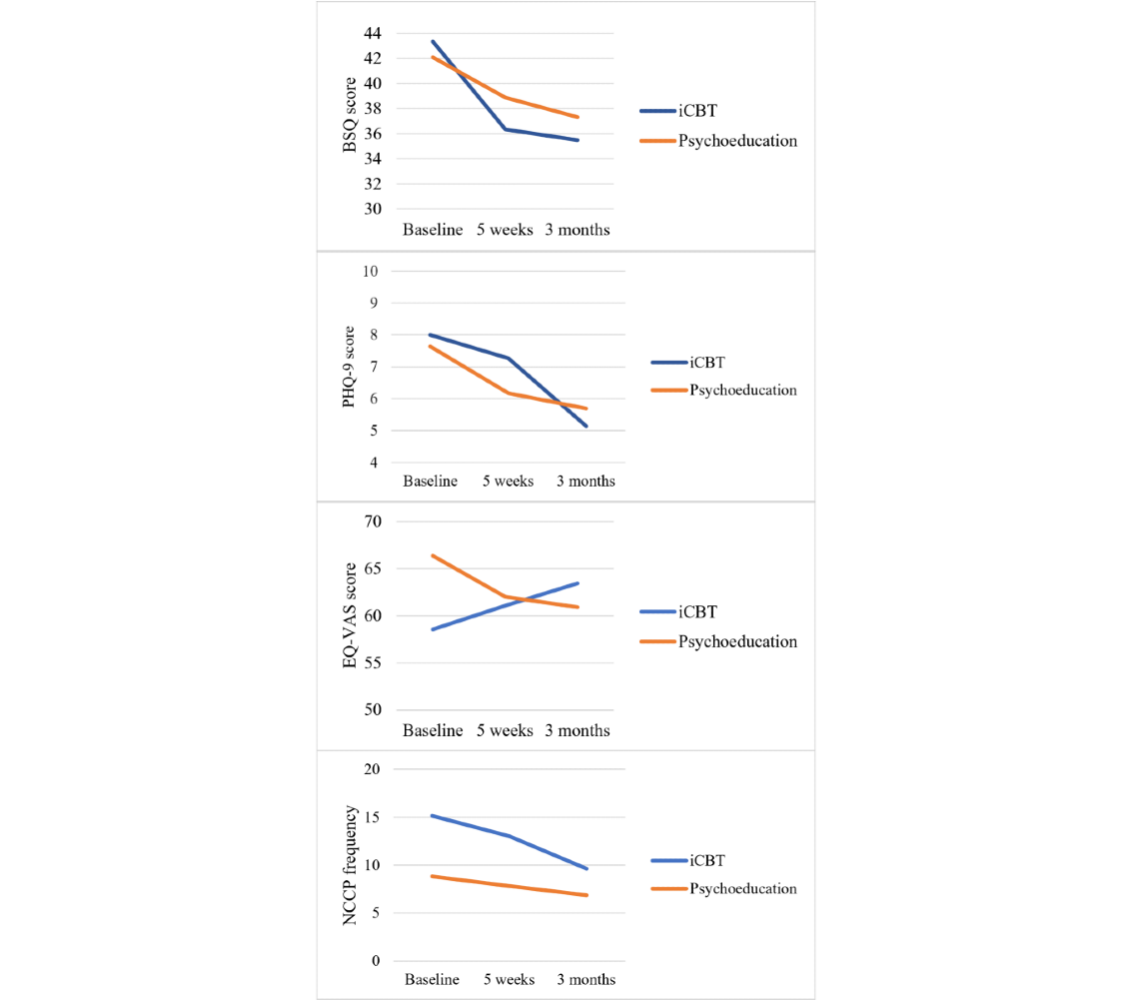
Changes in bodily sensations, depressive symptoms, EuroQol visual analog scale (EQ-VAS), and noncardiac chest pain (NCCP) frequency over time between internet-delivered cognitive behavioral therapy (iCBT) and psychoeducation groups. BSQ: Body Sensations Questionnaire; PHQ-9: Patient Health Questionnaire-9.

No significant interaction effect of time and group was found in any of the secondary outcomes (ie, fear of bodily sensations, depressive symptoms, HRQoL, and chest pain frequency) over the 3-month follow-up in the iCBT and psychoeducation groups (Table 2 and Figure 3). Table 3 displays the number of patients with depressive symptoms ≥10 in the iCBT and psychoeducation groups. In the iCBT group, only 11% (6/54) of patients reported depressive symptoms at the 3-month follow-up compared with 39% (21/54) of patients at baseline. For the psychoeducation group, of the 55 patients, the corresponding numbers were 9 (16%) patients at the 3-month follow-up and 13 (24%) at baseline. The effect sizes of the iCBT group on the secondary outcomes were trivial to moderate (Cohen *d* between 0.10 and 0.57) compared with those of the psychoeducation group. There were statistically significant differences regarding the time effect in bodily sensations at both 5-week and 3-month follow-ups (*P*<.001) and in depressive symptoms and chest pain frequency at the 3-month follow-up (*P*=.005) compared with baseline. Comparing the groups without considering the time effects, there were significant differences in HRQoL (*P*=.03) and chest pain frequency (*P*=.006) in favor of iCBT.

**Table 3:** Changes in the number of patients with depressive symptoms ≥10 between baseline and 3-month follow-up in the internet-delivered cognitive behavioral therapy (iCBT) and psychoeducation groups.

Measurement point	Patients with depressive symptoms, n (%)	
	iCBT (n=54)	Psychoeducation (n=55)
Baseline	21 (39)	13 (24)
5-week follow-up	15 (28)	9 (16)
3-month follow-up	6 (11)	9 (16)

## Discussion

### Principal Findings

To our knowledge, this is the first randomized controlled iCBT study targeting cardiac anxiety in patients with NCCP. In this study, we compared a 5-week–long nurse-led iCBT program comprising psychoeducation, mindfulness, and exposure to physical activity, with psychoeducation only to evaluate the effects on psychological distress, including cardiac anxiety, and other patient-reported outcomes in patients with NCCP. Our findings showed that iCBT was not superior to psychoeducation in decreasing cardiac anxiety in these patients. We found that one-third of the patients who received iCBT reported decreased anxiety levels compared with one-fourth of those who received psychoeducation, that is, a reliable change score of at least 11 points on the CAQ. Another important aspect is that only one of the patients in each group reported increased cardiac anxiety scores at the 3-month follow-up, indicating that the program was safe.

Although no significant interaction effect of time and group was found between the groups, iCBT seems to have better long-term effects on cardiac anxiety than psychoeducation, as within-group analysis showed that patients in the iCBT group had improved further at the 3-month follow-up (*P*=.04), which the psychoeducation group did not. In addition, patients who received iCBT tended to show long-term improvement in all reported variables (Figures 2 and 3), whereas those in the psychoeducation group did not change further. We also found a possible interaction effect of time and group regarding HRQoL in favor of the iCBT group with a moderate effect size. The patients improved over the measurement points (EQ-VAS increased from 58.5 at baseline to 65.5 at the 3-month follow-up), whereas the psychoeducation group deteriorated (EQ-VAS decreased from 66.4 at baseline to 61.5 at the 3-month follow-up).

These findings point to the fact that results related to changes in psychological distress and HRQoL may take more time to appear and that our intervention might be too short to detect such changes. A systematic review and meta-analysis by Reavell et al [51] showed a significant reduction in anxiety in long CBT interventions but not in short- to medium-term interventions compared with controls. These interventions were performed in patients with cardiovascular disease, and patients might need a longer time to adjust to their cardiac diagnosis and handle their anxiety compared with patients with NCCP. Previous studies in patients with NCCP using short interventions have reported inconsistent results [13,25,52,53]. Jonsbu et al [13] used a 3-session CBT intervention and reported significant effects in favor of CBT on fear of bodily sensations, avoidance of physical activity, depression, and some domains of HRQoL, and these effects lasted up to 12 months. However, cardiac anxiety was not studied. In a study by van Beek et al [25], positive effects on anxiety were found 24 weeks following a 6-session CBT intervention compared with usual treatment in patients with NCCP and panic disorder. Mulder et al [52] reported significantly lower health anxiety scores at 3 months but not at 12 months in patients who received 3 to 4 sessions of CBT compared with care as usual. The authors suggested booster sessions or longer interventions for more sustained improvement. Tyrer et al [53] failed to present any significant differences between iCBT and standard care at both 6- and 12-month follow-ups. Their CBT intervention comprised 4 to 10 sessions (mean 5.7) during a mean period of 14.3 weeks. They concluded that these results were mainly because of the study being underpowered. In an 8-year follow-up of CBT on health anxiety [54], the authors reported sustained improvement in health anxiety symptoms, especially among patients with cardiac diseases, and this improvement was greatest in nurse-led CBT. However, different length and follow-up periods for the interventions were used in these studies, which also used face-to-face CBT, compared with passive controls, which makes these studies difficult to compare with our study. This suggests that CBT is valuable to medical patients, including NCCP, with anxiety; however, the key is to find the right duration and type of CBT modality.

Furthermore, we believe that we could not find significant differences as iCBT was compared with an active control. In addition to the possible placebo effect of being in a trial and having their symptoms recognized, receiving psychoeducation, which our control group did, seems to have had effects on both primary and secondary outcomes, which could explain the minor and nonsignificant differences between the iCBT and psychoeducation groups [41]. Psychoeducation makes it easier for patients to understand what therapeutic tools to use and facilitates the motivation for behavior change and finding different ways of thinking in relation to their condition [55]. This can be compared with other studies comparing iCBT with usual care or waiting list instead of an active control and reporting significant improvement in anxiety and depression in favor of iCBT [25,30]. Studies with passive controls, such as waiting list or standard care, often report better outcomes in favor of the intervention compared with those with active controls [56]. An important aspect to consider is how long the effects of psychoeducation last and whether more psychological tools, such as cognitive restructuring and behavioral change strategies, are needed to maintain long-term results.

In the iCBT group, a larger proportion (21/54, 39% vs 13/55, 24%) of patients had at least moderate levels of depressive symptoms at baseline, although the mean values in the group did not differ significantly from those in the psychoeducation group. Although the study was not designed to target depressive symptoms, the iCBT program had significant effects on depressive symptoms in most of these patients, as 71% (15/21) reported lower scores on the PHQ-9 (ie, <10) than was reported in patients who received psychoeducation, in which only 31% (4/13) reduced depressive symptom levels. On the basis of our previous findings revealing that depressive symptoms have strong effects on cardiac anxiety in patients with NCCP, we believe that the effects of iCBT targeting cardiac anxiety might have been limited by the higher proportion of patients with depressive symptoms in this group [57]. Another affecting aspect could be that the length of our program was too short to lead to improvement, as comorbid depressive symptoms need longer treatments than if patients only have anxiety [58].

### Strengths and Limitations

Of the 824 patients assessed, only 109 (13.2%) took part in the study. This could indicate that the population in this study may not be representative of patients with NCCP in general. Patients were included based on high levels of anxiety, as the program was designed to target anxiety. As the CAQ lacks a cutoff score for anxiety, we used the median value in our previous study. This might have been too high, as many patients who perceived themselves to have cardiac anxiety did not come up to this cutoff and were, therefore, excluded from the study. As we had little missing values, we chose to report the original data; however, we also provided a mixed model analysis with multiple imputed data to ensure the accuracy of our results. Regardless of the type of statistical analysis, our result proved to remain. Some patients could not join the study because of a lack of computer or internet access or perceive themselves as having difficulties in handling such interventions. However, we are aware that internet interventions cannot fit all patients, partly because of technical aspects but mainly because of preferences for face-to-face therapies [30]. We had difficulties recruiting patients to the study and had to expand our recruitment sites during the study. This could be as many patients with NCCP are not motivated to receive psychological treatment as they perceive their pain to be physical. As stated previously, the iCBT program accounted for the main part of the treatment effect, and the guidance and feedback part mainly aimed at confirming patients’ work and progress and encouraging them to reflect on different issues and take on new challenges. The guidance and feedback were mainly provided by the first author (GM) and, in some cases, by the second author (MEL), who was mentored by the first author. This indicates that feedback can be provided by different health care professionals with a short introduction to iCBT.

### Conclusions

iCBT was not superior to psychoeducation in decreasing cardiac anxiety in patients with NCCP. However, iCBT tends to have better long-term effects on psychological distress, including cardiac anxiety, HRQoL, and NCCP frequency than psychoeducation. Better conclusions may be drawn based on further follow-ups.

## Appendix

Multimedia Appendix 1 CONSORT-eHEALTH (V1.6.1)

## Abbreviations

BSQ: Body Sensations Questionnaire
CAQ: Cardiac Anxiety Questionnaire
CBT: cognitive behavioral therapy
EQ-VAS: EuroQol visual analog scale
HRQoL: health-related quality of life
iCBT: internet-delivered cognitive behavioral therapy
NCCP: noncardiac chest pain
PHQ-9: Patient Health Questionnaire-9
